# Color Doppler imaging characteristics of RPE neoplasms: A study of 17 cases

**DOI:** 10.3389/fopht.2022.758851

**Published:** 2022-11-24

**Authors:** Nan Zhou, Wenli Yang, Wenbin Wei

**Affiliations:** Beijing Tongren Eye Center, Beijing Key Laboratory of Intraocular Tumor Diagnosis and Treatment, Medical Artificial Intelligence Research and Verification Laboratory of the Ministry of Industry and Information Technology, Beijing Tongren Hospital, Capital Medical University, Beijing, China

**Keywords:** “black-linear” sign, RPE adenoma/adenocarcinoma, color Doppler imaging, diagnosis, uveal melanoma

## Abstract

**Purpose:**

To report the color Doppler imaging (CDI) features of 17 patients with retinal pigment epithelium (RPE) neoplasms.

**Design:**

Retrospective, observational case series.

**Participants:**

Seventeen patients with RPE adenoma/adenocarcinoma.

**Methods:**

Contrast-enhanced color Doppler ultrasonography (CDU) was performed in 16 patients with RPE adenoma and in one patient with adenocarcinoma.

**Result:**

All 17 RPE neoplasms showed well-defined margins. An oval mass was observed in 11 tumors, a lenticular-shaped mass in four tumors, and a flat mass in two tumors. On CDI, RPE lesions showed homogeneous reflectivity of moderate intensity with no choroidal excavation. After the contrast-enhanced administration of sulfur hexafluoride, mild enhancement was identified in six tumors and moderate enhancement in 11 tumors. In all 17 RPE neoplasms, contrast-enhanced CDU time–intensity curves (TICs) exhibited fast fill-in and slow washout-shaped curves with the dominant vessel within RPE lesions. A “black-linear” sign (defined as a low signal intensity-linear zone located between the tumor and enhanced choroid on CDI) was noted in all 17 patients with RPE neoplasms.

**Conclusions:**

This preliminary study noted a “black-linear” sign on CDI of RPE lesions for the first time. The novel sign may be a diagnostic characteristic of RPE neoplasms and may help to distinguish this rare entity from uveal melanomas (UMs).

## Introduction

Retinal pigment epithelium (RPE) neoplasms are extremely rare intraocular tumors (1–3). Most of them are benign RPE adenomas. Clinically, RPE adenomas may simulate uveal melanomas (UMs) (4, 5), and therefore, may easily be misdiagnosed. Our previous studies, along with those of Shields, have summarized the clinical features of RPE adenomas and the associated clinical criteria that may help to differentiate RPE adenomas from UMs (4, 5). The management of RPE adenomas and UMs is completely different. Most cases of UMs have been treated with plaque radiotherapy and RPE adenomas/adenocarcinomas with local resection by a 23- to 25-gauge microinvasive vitrectomy (5). Differentiating the two entities is crucial in treatment discussions and decision-making.

The published literature on CDI features of RPE neoplasms is sparse. Most consist of isolated cases or small case series, and the ordinary ultrasonographic findings could not differentiate RPE neoplasms from UMs (5). RPE lesions demonstrate masses with relatively consistent reflectivity of moderate intensity with arterial blood signals within the tumor. CDI findings reported in these studies resemble those of UMs.

The present study reports the CDI features of 16 RPE adenomas and one RPE adenocarcinoma using advanced contrast-enhanced color Doppler ultrasonography (CDU). The observations of a new sign (i.e., a “black-linear” sign) described here may help to separate this rare entity from UM.

## Methods

### Case series

This retrospective observational study included 16 patients with RPE adenoma and one patient with adenocarcinoma who were followed from August 2007 to April 2020 at the Beijing Institute of Ophthalmology, Beijing Tongren Hospital (Capital Medical University, Beijing Key Laboratory of Intraocular Tumor Diagnosis and Treatment, China). The study followed the tenets of the 1964 Declaration of Helsinki and its ethical standards. The study was approved by the Medical Ethics Committee of the Beijing Tongren Hospital, and written informed consent was obtained from all participants. All patients with RPE neoplasms underwent standard clinical evaluation, including best-corrected visual acuity (BCVA) measurement with Snellen charts, dilated fundus examination, slit lamp examination and imaging with fundus color photography, contrast-enhanced CDU, indocyanine green angiography (ICGA), and fluorescein angiography (FA), and optical coherence tomography (OCTA) images. All 17 cases of RPE neoplasm underwent local surgical resection by microinvasive (23- 25-gauge) sutureless vitrectomy, and the clinical diagnosis was confirmed by pathohistological analysis.

### Contrast-enhanced color Doppler imaging protocols

Available contrast-enhanced CDI data were retrieved for all 17 patients. All patients underwent ocular color Doppler imaging on MyLab90 Color Doppler (Esaote, S.p.A., Italy) ultrasonography, and the probe frequency was (3–9) × 106 Hz. The power remained at about 20% while performing color Doppler flow imaging, and the mechanical index was maintained at 0.4 or less. Two-dimensional ultrasound was applied to observe the locations and acoustic characteristics of the lesions. Blood flow within the lesions was visualized by color Doppler flow imaging. Sulfur hexafluoride (SonoVue, Braco, Imaging B.V., Switzerland) was applied as contrast agent. Post-contrast-tuned imaging (CnTI) was acquired after intravenous bolus injection of 2.4 ml of sulfur hexafluoride and washed with 5 ml of physiological saline.

SonoLiver 1.1 (TomTec Imaging Systems, Munich, Germany) was used to quantitatively analyze the filling process and the contrast-enhanced CDU results. The parameters of the lesions and control tissues were as follows: maximum intensity (MI), rising time (RT), time to peak (TTP), and mean transit time (MTT).

### Contrast-enhanced color Doppler imaging analysis

CD imaging findings of RPE adenomas were evaluated with emphasis on the tumor location, basal diameter, margin, shape, height, signal reflectivity of intensity compared with the normal orbital tissue, and homogeneity and degree of enhancement of the lesions. Signal intensity on post-contrast-enhanced CDI higher than that of the normal orbital tissue was defined as dynamic enhancement; the signal intensity lower than that of the normal orbital tissue was defined as mild enhancement; and the signal intensity equal to that of the normal orbital tissue was defined as moderate enhancement.

**Figure 1:**
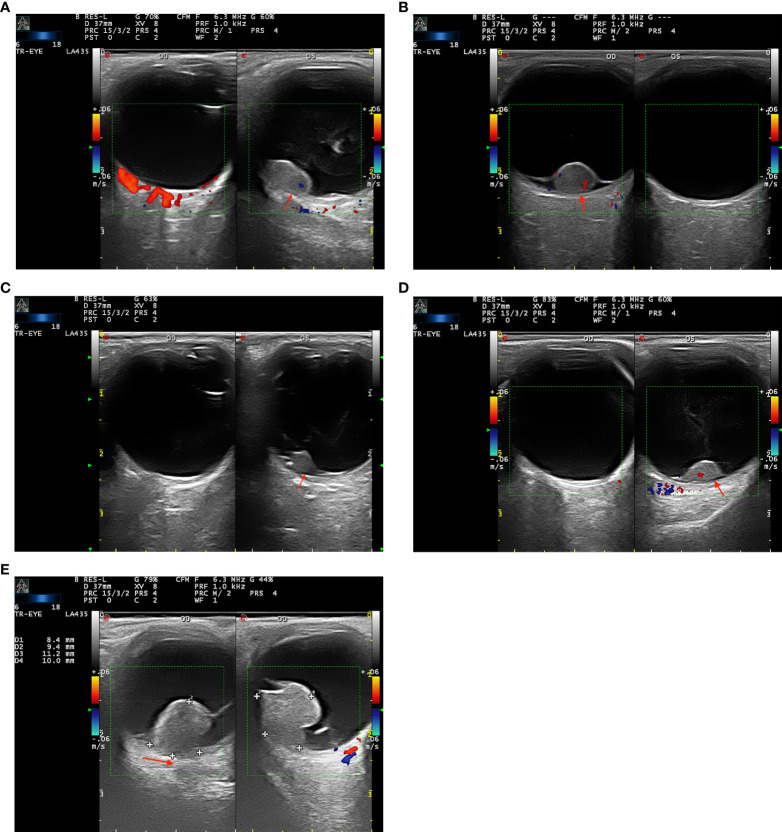
**(A–D)** Color Doppler imaging (CDI) shows the “black-linear” sign in three patients with retinal pigment epithelium (RPE) adenoma **(A–C)** and one patient with (RPE) adenocarcinoma. The well-defined lenticular, oval-shaped masses manifest moderate signal intensity with no choroidal excavation and bulging posterior choroid. The lesions show mild or moderate enhancement and present with the “black-linear” sign (a low signal linear shadow between the lesion and the marked enhanced choroid, red arrow). **(E)** A 57-year-old woman with uveal melanoma (UM) in the right eye. A mushroom-shaped mass shows a moderate signal intensity with choroidal excavation and bulging posterior choroid sign (red arrow).

A slim- and low-signal linear zone located between the tumor lesion and the significantly enhanced choroid was defined as a “black-linear” sign on post-contrast-enhanced CDI (**Figures 1A–D**). This novel sign was not described in previous CDI studies. All CD imaging findings of the tumors were reviewed and evaluated by an ultrasonographer (W.L.Y., with 10 years of experience) and confirmed by an ophthalmologist (W.B.W., with 20 years of experience).

The blood flow of the dominant vessel of RPE neoplasms was shown in either red or blue, and the direction of the blood flow was dependent on the direction of the flow to the transducer. Doppler spectral analysis was done to distinguish between pulsatile arterial flow and the more continuous or minimally pulsatile venous flow. Data quantification was performed by frequency spectrum analysis. The relationships between concentration and the filling time of the contrast agent (sulfur hexafluoride) in different tissues were quantitatively analyzed. The classification of the contrast-enhanced CDU-TICs used in the present study was analyzed as washout-shaped curves, including fast fill-in to fast washout of the contrast agent and fast fill-in to slow washout of the contrast agent, respectively.

## Results

All 17 patients (nine men and eight women) underwent CDU examination. The mean age was 43 years (median, 40; range, 25–63). A summary of the clinical information, surgical process, histopathological findings, and follow-up results is provided in **Table 1**. The ultrasonographic features of these 17 patients with RPE neoplasm are shown in **Table 2**.

**Table 1:** Demographic data, initial site, outcome, and period of follow-up for patients with adenoma/adenocarcinoma of the retinal pigment epithelium (RPE), who were treated with local resection by microinvasive (23–25 gauge) vitrectomy.

Patient no./Age (years)/Sex/Ethnicity	Year of diagnosis	Location	Involved eye(s)	Associated Findings	Management	Histopathologic diagnosis	Visual acuity		Follow-up after local resection (years)
							Before treatment	After treatment	
1/31/M/A	2007	MP	R	Pi, RFV IE	Vitrectomy, resection	Adenoma	20/200	20/200	13
2/57/M/A	2013	MP	R	Pi, RFV IE	Vitrectomy, resection	Adenoma	20/50	20/66	7
3/39/M/A	2013	MP	L	Pi, ERD SWR, RFV	Vitrectomy, resection	Adenoma	20/200	20/200	7
4/56/F/A	2014	M	R	Pi, RFV MD, IE	Vitrectomy, resection	Adenoma	20/400	20/400	6
5/25/M/A	2018	P	L	Pi, VH RFV, IE	Vitrectomy, resection	Adenoma	20/400	20/66	2
6/55/F/A	2018	MP	L	Pi, IE RFV	Vitrectomy, resection	Adenoma	20/66	20/66	2
7/41/M/A	2019	MP	L	Pi, ME RFV	Vitrectomy, resection	Adenoma	20/66	20/66	1.5
8/35/F/A	2017	JP	R	Pi, MD RFV	Vitrectomy, resection	Adenoma	CF	ant peroxidase	3
9/35/F/A	2017	JP	R	Pi, ERD MD	Vitrectomy, resection	Adenoma	20/400	20/400	3
10/40/F/A	2018	P	R	ERD, IE Nonpi, RFV	Vitrectomy, resection	Adenoma	20/400	20/400	2
11/33/M/A	2018	MP	L	Pi, RFV IE	Vitrectomy, resection	Adenocarcinoma	20/33	20/33	2
12/58/F/A	2019	P	L	Pi, RFV IE	Vitrectomy, resection	Adenoma	20/33	20/33	1.5
13/63/F/A	2018	P	R	Pi, Nonpi	Vitrectomy, resection	Adenoma	20/25	20/33	2
14/40/F/A	2019	M	R	Pi, MD RFV, IE	Vitrectomy, resection	Adenoma	CF	CF	1.5
15/38/F/A	2007	MP	R	MD, IE SWR, Nonpi	Vitrectomy, resection	Adenoma	CF	CF	13
16/51/M/A	2007	JP	R	Nonpi	Vitrectomy, resection	Adenoma	CF	CF	13
17/36/F/A	2019	MP	R	Pi, RFV, IE	Vitrectomy, resection	Adenoma	20/50	20/50	1

F, female; M, male; R, right; L, left; CF, counting fingers; A, Asian; JP, juxtapapillary; P, peripheral; MP, midperipheral; M, macula; RFV, retinal feeder vessel; IE, intraretinal exudation; SWR, surface wrinkling retinopathy; ERD, exudative retinal detachment; VH, vitreous hemorrhage; ME, macular edema; MD, macular detachment; Nonpi, Nonpigmented; and Pi, pigmented.

**Table 2:** Color Doppler (CD) imaging features of the 17 retinal pigment epithelium (RPE) neoplasms.

Patient no./Age (years)/Sex/Ethnicity	Tumor shape	Size (mm)	Signal intensity	Black-linear sign	Choroidal excavation	Enhancement
1**/**31/M/A	Oval	6.5*8.5*9.3	Moderate	Y	N	Moderate
2**/**57/M/A	Oval	6.9*4.3*7.5	Moderate	Y	N	Moderate
3**/**39/M/A	Oval	7.3*4.4*7.2	Moderate	Y	N	Moderate
4**/**56/F/A	Flat	5.3*2.3*5.1	Moderate	Y	N	Mild
5**/**25/M/A	Oval	6.5*5.0*8.1	Moderate	Y	N	Mild
6**/**55/F/A	Oval	5.9*3.5*6.2	Moderate	Y	N	Moderate
7**/**41/M/A	Lenticular	7.6*4.1*8.6	Moderate	Y	N	Mild
8**/**35/F/A	Lentiform	5.5*5.9*5.2	Moderate	Y	N	Moderate
9**/**35/F/A	Oval	5.7*4.4*5.3	Moderate	Y	N	Moderate
10**/**40/F/A	Lentiform	6.1*3.2*5.0	Moderate	Y	N	Moderate
11**/**33/M/A	Lentiform	6.6*3.2*6.6	Isointense	Y	N	Mild
12**/**58/F/A	Oval	7.5*1.8*5.5	Moderate	Y	N	Moderate
13**/**63/F/A	Oval	1.7*3.2*2.4	Moderate	Y	N	Mild
14**/**40/F/A	Flat	7.0*2.1*8.0	Moderate	Y	N	Moderate
15**/**38/F/A	Oval	9.3*8.0*6.6	Moderate	Y	N	Moderate
16**/**51/M/A	Oval	5.7*5.2*7.1	Moderate	Y	N	Mild
17**/**36/F/A	Oval	11.6*3.9*9.3	Moderate	Y	N	Moderate

Y, Yes; N, No.

**Figure 2:**
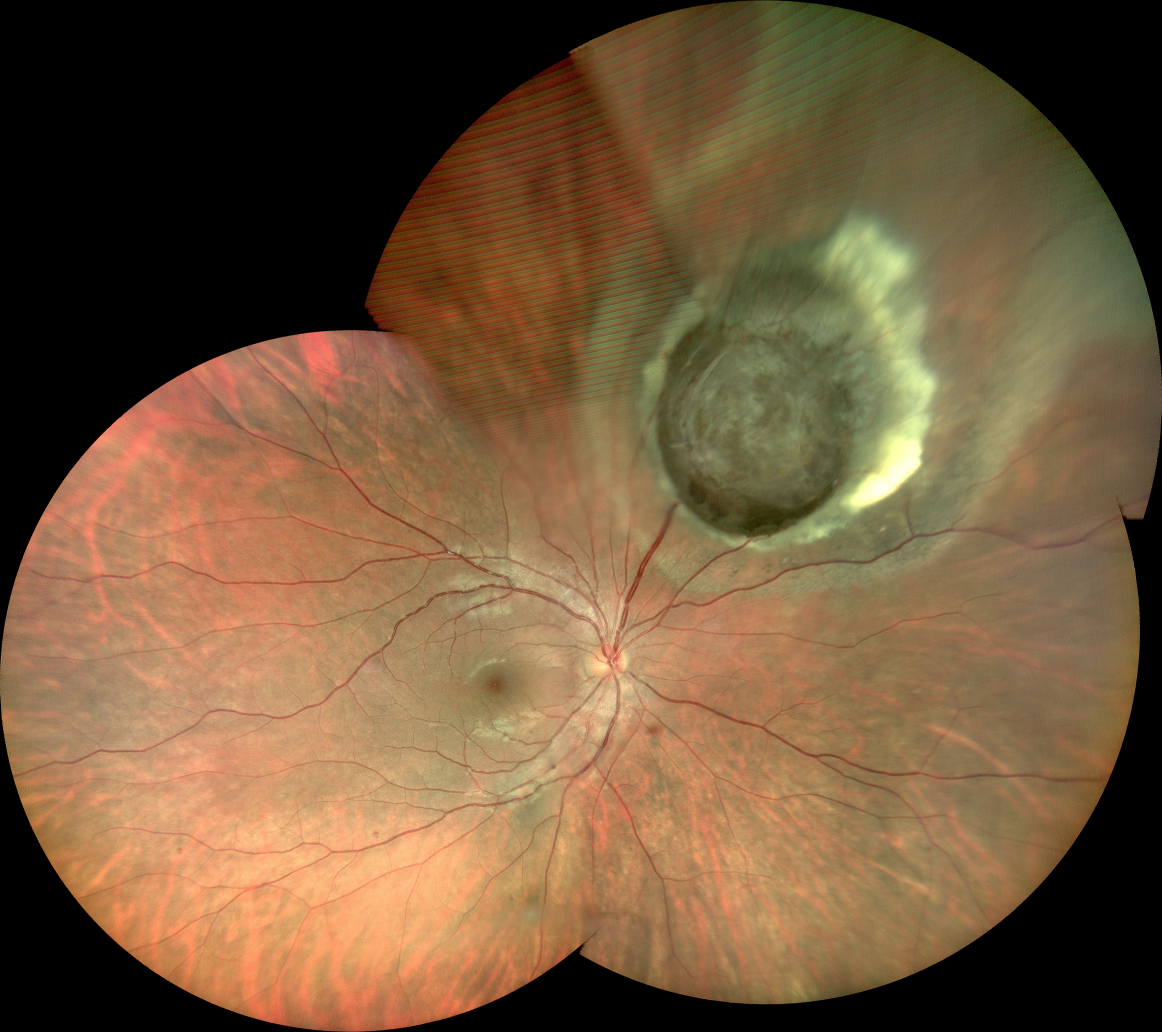
Superonasal pigmented tumor with prominent, dilated, and tortuous feeding artery and draining vein, surrounding yellow lipid exudation adjacent to the neoplasms (Patient 17).

**Figure 3:**
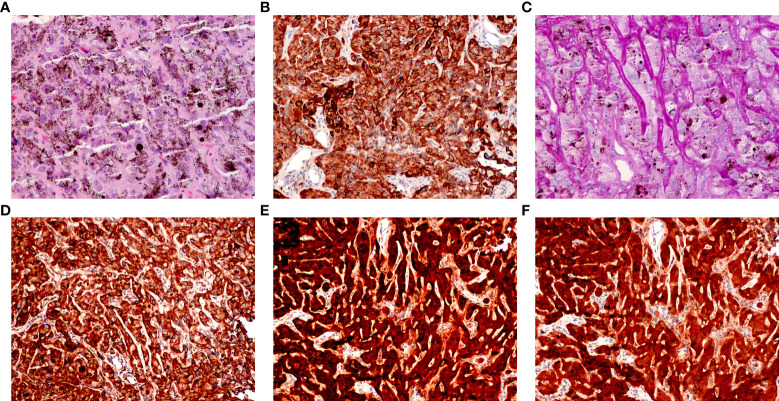
Pathological features of retinal pigment epithelium (RPE) adenoma (Patient 5). **(A)** Photomicrograph of amelanotic tumor cells, showing a cord-like arrangement, resembling an epithelium (HE, ×100). **(B)** Tumor cells, showing positive immunoreactivity for CK, **(C)** PAS (periodic acid–Schiff, × 100), **(D)** vimentin, **(E)** S-100, and **(F)** NSE (peroxidase–antiperoxidase, × 100).

The tumor was located in the juxtapapillary area in one patient, in the macular area in two patients, and in the peripheral fundus in the remaining 14 patients. The neoplasms were dark-black pigmented in 14 patients and nonpigmented in three patients (patients 10, 15, and 16). The dominantly dilated, tortuous feeder, and drainer blood vessels were obvious, and were seen entering and exiting the tumors in 16 of the 17 patients (**Figure 2**). Different amounts of yellow intraretinal exudation were also observed adjacent to the neoplasms in 16 of the 17 patients, and one of them (patient 10) had a large, secondary exudative retinal detachment. In addition, two patients had surface wrinkling retinopathy, and three patients had exudative macular detachment. All of these changes were secondary to RPE neoplasms. All 17 patients were managed with 23- to 25-gauge microinvasive vitrectomy for the local resection of intraocular tumors and reconstruction of the globe. The pathohistological analysis revealed that the tumor cells had a slightly pleomorphic round or oval nucleus Hemotoxin & Eosin (HE) and were positive for S-100, neuron specific enolase (NSE), cytokeratin (CK), periodic acid–Schiff (PAS), and vimentin (**Figures 3A–F**).

**Table 2** shows the CDI features of all 17 patients with RPE neoplasms. An oval mass was seen in 11 tumors, lenticular-shaped in four tumors, and flat in two tumors. The mean largest basal diameter of RPE neoplasms was 6.63 mm (median, 6.5; range, 1.7–11.6), and the mean tumor thickness was 4.3 mm (median, 4.1; range, 1.8–8.5) (**Table 2**), respectively. The basal dimensions of RPE neoplasms ranged from 1.7 × 3.2 × 2.4 to 11.6 × 3.9 × 9.3 mm^3^. All tumors revealed well-defined margins and relatively homogeneous reflectivity.

**Figure 4:**
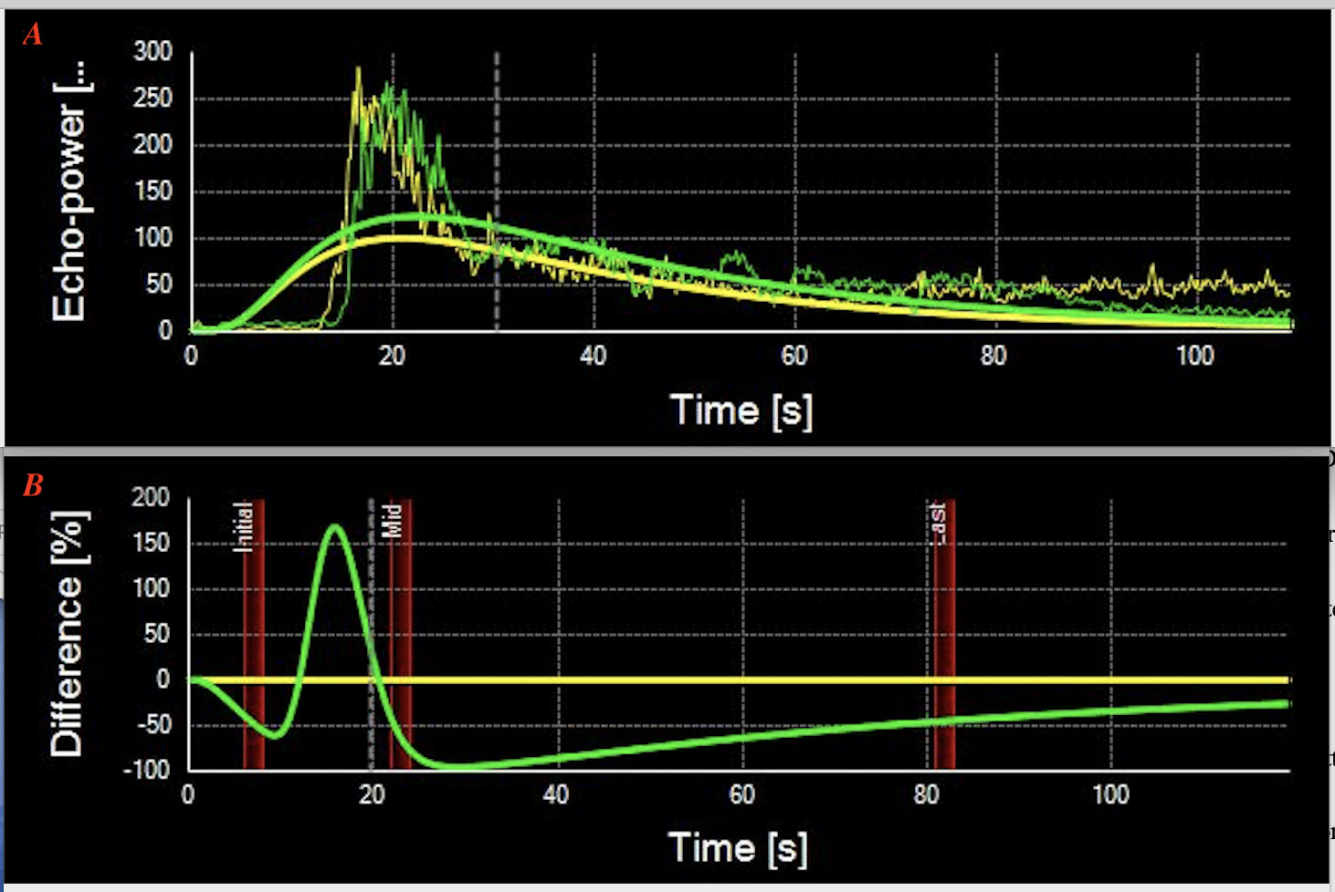
**(A)** Contrast-enhanced color Doppler ultrasonography-time–intensity curves (CDU-TICs) of the retinal pigment epithelium (RPE) neoplasms present a specific fast fill-in and slow washout-shaped curves (Patient 9). **(B)** Contrast-enhanced CDU-TICs of the uveal melanoma present with fast fill-in to fast washout-shaped curves.

All 17 RPE neoplasms had relatively consistent reflectivity of moderate signal intensity and no choroidal excavation. After contrast-enhanced administration of sulfur hexafluoride, mild enhancement was identified in six tumors and moderate enhancement in 11 tumors. The “black-linear” sign was observed in all 17 patients on the post-contrast CD imaging, and no extra-scleral extension of the neoplasm was demonstrated in any patient. The length of the “black-linear” sign was less than the tumor basal diameter in all cases. The contrast-enhanced CDU-TICs presented the fast fill-in and slow washout-shaped curves (**Figure 4A**).

## Discussion

In recent years, additional reviews about RPE adenomas have been published, and we have a hypothesis that RPE adenomas have approximately the same incidence as UMs. However, differentiation between RPE adenomas and UMs is important and is still challenging because these lesions are benign, and enucleation is not necessarily performed on such benign lesions (3, 6–8). RPE adenomas often have similar characteristics to UMs under indirect ophthalmoscopy, MRI, and CDI (1, 3–5). Under ophthalmoscopy, RPE adenomas appear as dark brown or black masses, similar to UMs (3–7). On MRI, RPE adenomas are hyperintense on T1WI and hypointense on T2WI, similar to the paramagnetic effects of melanin produced by UMs (9). In addition, most RPE adenomas appear as solid, dome-shaped, well-defined masses with the “black-linear” sign on post-contrast T1WI (9); the “black-linear” sign may provide some clues to diagnosis.

Published literature on the CD imaging features of RPE adenomas is sparse. In the current study, RPE adenomas showed relatively homogeneous reflectivity of moderate intensity on CDI. Most RPE adenomas could be seen with dark pigmentation, which may have similar ultrasonic signal reflective effects to those seen in UMs. Based on signal intensity changes alone, CD imaging cannot differentiate RPE adenomas from UMs.

Although very similar to UMs, morphology may provide a means to distinguish RPE adenomas from UMs. UMs often demonstrate the characteristic mushroom shape and have been occasionally associated with extraocular extension (3–5, 10). However, no mushroom shape was found in our 17 RPE neoplasms. We noted that RPE adenomas often have an oval configuration, with the absence of extraocular extension. These features may serve to distinguish the two entities. Previous studies reported the typical features of UMs on CDI, the choroidal excavation, and the finding of an indentation of the abnormally rough, concave choroidal outline, caused by a choroidal infiltration (11–13). These signs were not observed in patients with RPE neoplasms in our case series. Therefore, the presence or absence of choroidal excavation and bulging posterior choroid sign may also help in the differential diagnosis (**Figure 1E**).

Post-contrast CDI demonstrated mild to moderate degrees of enhancement on all of the 17 patients with RPE neoplasms. The TICs showed a specific fast wash-in and slow washout on 17 contrast-enhanced CDI-available tumors, compared with the fast wash-in to fast washout curves on UMs (**Figure 4B**). However, on contrast-enhanced CD imaging, we noted a “black-linear” sign that may prove to be specific to RPE neoplasms. This sign appeared as a low-signal linear zone located between the tumor and the enhanced wall of the eyeball. It is characterized by a single smooth, continuous, hyperintense, and enhanced rim under the low-signal linear zone, corresponding to the choroid. In addition, the resolution of CDU is in millimeters. Therefore, the “black-linear” sign on CD images will not be mistaken for the minor subretinal fluid (SRF) of lesions. A large amount of shifting SRF can be visible on the CD images and is often located around the tumor lesion or on the inferior fundus (due to the effect of gravity); however, the B-scan of optical coherence tomography/angiography (OCT/A, resolution is in micrometers) can show a small amount of SRF in the base of the lesions. This sign has never been reported before, and to the best of our knowledge was not observed in any other neoplasms in this location. Regarding the UMs that originated from the uveal or choroid, they appeared as mushroom-shaped configurations once they broke the Bruch’s membrane during tumor growth. Adenomas that originated from RPE appeared as abrupt elevated oval masses. They lacked the adjacent base of choroidal tumors seen with most UMs. The different origination of the two entities would be the cause of the “black-linear” sign on CDI.

In the present study, we found the diagnostic criteria for CDI features of RPE adenomas to be (1) the novel dark-linear sign; (2) the lack of choroidal excavation; (3) the lack of choroidal infiltration signs; and (4) the fast fill-in and slow washout-shaped curves present in the contrast-enhanced CDU-TICs. These characteristics on CDI may help to distinguish RPE adenomas from UMs.

The limitations of this study should be mentioned. The first limitation of this analysis was its retrospective nature and single-center research. Second, the sample of 17 patients with RPE neoplasms was relatively small. Third, there was a lack of genetic testing analysis. A future study of the genetic composition of RPE neoplasms will help to achieve accurate diagnosis.

## Conclusion

RPE adenoma and adenocarcinoma can simulate UMs, with similar clinical and CDI features. While prior studies have described CDI features of UMs that may be absent in RPE neoplasms, such as choroidal excavation and bulging posterior choroid, fewer studies have investigated CDI features specific to RPE tumors. In this study, we have described a novel CDI feature of RPE tumors, named the “black-linear” sign. This sign, characterized by a slim, low-signal linear zone between the tumor and the enhanced choroid, may be a unique diagnostic feature of tumors located in the RPE.

## Data availability statement

The original contributions presented in the study are included in the article/Supplementary Material. Further inquiries can be directed to the corresponding author.

## Ethics statement

Studies involving human participants were reviewed and approved by the Beijing Tongren Hospital Ethics Committee. The patients/participants provided their written informed consent to participate in this study. Written informed consent was obtained from the individual(s) for the publication of any potentially identifiable images or data included in this article.

## Author contributions

WW: Examination of the patients, interpretation of the results, writing of the manuscript. NZ: Interpretation of the results and writing/reviewing of the manuscript. WY: Interpretation of the results and reviewing of the manuscript. All authors contributed to the article and approved the submitted version.

## Funding

The National Natural Science Foundation of China (No. 81272981) and the Beijing Natural Science Foundation (No. 7151003) provided financial support.

## Conflict of interest

The authors declare that the research was conducted in the absence of any commercial or financial relationships that could be construed as a potential conflict of interest.

## Publisher’s note

All claims expressed in this article are solely those of the authors and do not necessarily represent those of their affiliated organizations, or those of the publisher, the editors and the reviewers. Any product that may be evaluated in this article, or claim that may be made by its manufacturer, is not guaranteed or endorsed by the publisher.
